# Interdisciplinary Management of Gingivitis Artefacta Major: A Case Series

**DOI:** 10.1155/2015/678504

**Published:** 2015-11-18

**Authors:** Naina Pattnaik, Anurag Satpathy, Rinkee Mohanty, Rashmita Nayak, Surjeet Sahoo

**Affiliations:** ^1^Department of Periodontics and Oral Implantology, Institute of Dental Sciences, Siksha ‘O' Anusandhan University, Khandagiri Square, Bhubaneswar, Odisha 751003, India; ^2^Department of Psychiatry, Institute of Medical Sciences & SUM Hospital, Siksha ‘O' Anusandhan University, Khandagiri Square, Bhubaneswar, Odisha 751003, India

## Abstract

Cases described here discuss interdisciplinary (periodontal and behavioral) approach in the management of rare and difficult to diagnose self-inflicted injuries of gingiva such as gingivitis artefacta major. Self-inflicted injuries to the gingiva are rare and their management by periodontal therapy alone is inadequate. Proper management of this condition requires early detection and effective psychological treatment through behavioral therapy in addition to the treatment of dental lesion. Three male patients in their twenties presented with traumatic injuries of gingiva with history of self-injury and underlying emotional disturbances. Following basic periodontal intervention, their self-inflicting behavior was confirmed on psychiatric consultation. All of them underwent cognitive behavior therapy and were able to successfully curb their self-inflicting behavior prior to any definitive dental procedures. These cases illustrate the essentiality of behavioral intervention in addition to periodontal procedures in the management of such lesions.

## 1. Introduction

Self-injurious behavior (SIB) has been described as an act of unassisted and deliberate injury to one's own body which is severe enough to cause tissue damage not including those with a conscious suicidal intent [1]. Although the traumatic injuries of the gingiva (accidental, iatrogenic, and factitious traumatic lesions) were included in the recent classification (AAP 1999) [2], SIB pertaining to periodontal tissues has been reported separately [3–6] and also termed as gingivitis artefacta.

Gingivitis artefacta is classified into three types [7]: Type A: injuries superimposed upon a preexisting lesion (or irritation), Type B: injuries secondary to another established behavior (such as thumb sucking), and Type C: injuries of unknown or complex etiology (often based on some emotional disturbance or psychological illness). This classification was further modified by Stewart [8] as minor: superficial gingival lesions resulting from rubbing or picking the gingiva with fingernails or sharp objects and major: severe and more widespread lesions with deeply entrenched habit.

In comparison to the minor form, gingivitis artefacta major is relatively rare and difficult to diagnose. It can be easily missed as a routine laceration or trauma since the clinical features do not differ often leading to recurrence and failure of corrective periodontal surgeries. These lesions are a physical manifestation of an underlying emotional disorder. Therefore, management of such cases requires early detection of underlying psychological state of the patient and should be referred for expert medical attention for effective psychological treatment through behavioral therapy in addition to the treatment of gingival lesion [9, 10]. Behavioral intervention may be considered as one of the first therapeutic options owing to its favorable outcome with little adverse effects as compared to pharmacological treatment. These conditions are prone to repeated failures in absence of thorough psychological assessment and treatment causing irreversible dental morbidity.

To our knowledge, very few cases of gingivitis artefacta major have been reported previously and none have elaborated the interdisciplinary approach. The purpose of this case series is to present the successful interdisciplinary (periodontal and cognitive behavioral) approach in the management of gingivitis artefacta major.

## 2. Case Reports

### 2.1. Case  1

**Figure 1:**
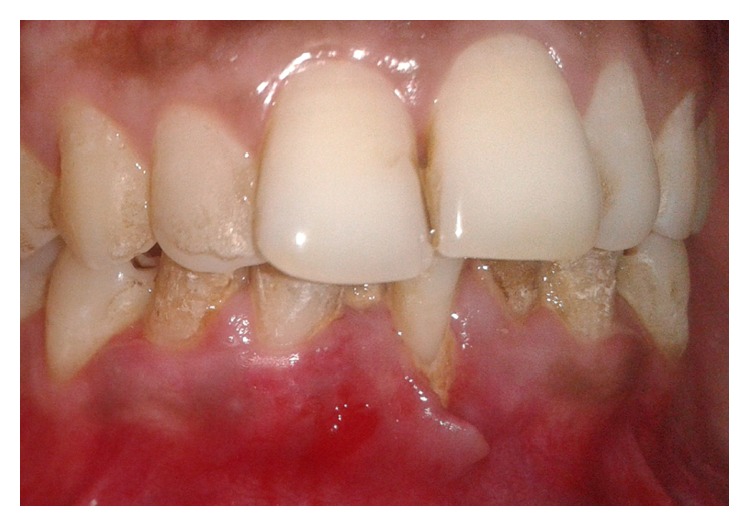
Case  1. Gingival clefting and recession due to scratching with fingernail in relation to tooth #41.

A 25-year-old male presented to the Department of Periodontics and Oral Implantology, Institute of Dental Sciences, Bhubaneswar, with a chief complaint of bleeding and pain in gums in the mandibular anterior region two weeks back. He admitted to have been scratching in the same region frequently with his fingernails on having sensitivity and irritation (Figure 1) which gave him a temporary relief. Occasionally, he used a matchstick in the same area till bleeding occurred and he felt relieved. On asking he revealed that he had lost his job four months back and was quite disturbed for the same reason. He further confided that he was feeling low and was depressed. Physically, he appeared emaciated and weak. His medical history was noncontributory and he was not currently under any medications.

Intraoral examination revealed erythematous gingiva in the area of chief complaint which bled profusely on probing. Also, it was partially detached with an irregular border giving a cleft-like appearance extending beyond the mucogingival junction in relation to mandibular right central incisor. He maintained poor oral hygiene with evidence of extrinsic stains, plaque, and calculus. No other pathological changes were seen in the oral cavity. He was then referred to the Department of Psychiatry, IMS and SUM Hospital, Bhubaneswar, where his SIB was confirmed. Therefore, a diagnosis of gingivitis artefacta major was made based on his history and clinical findings.

#### 2.1.1. Management

The patient was presented with, and accepted, a treatment plan which included periodontal and behavior management. A written informed consent was obtained. Periodontal treatment included scaling and root planing (SRP) with instructions for maintenance of oral hygiene. The patient was asked to trim his fingernail and to refrain from scratching or picking in the concerned area. He was prescribed 0.2% chlorhexidine gluconate containing mouthwash (Hexidine, ICPA Health Products Limited, Ankleshwar, India) to be used twice daily for a week. A local anesthetic gel containing 20% Benzocaine (Mucopain, ICPA Health Products Limited, Ankleshwar, India) was prescribed for symptomatic relief to be applied on the affected area for 1 week.

For behavioral management, he was put under cognitive behavioral therapy (CBT) with a trained therapist in the Department of Psychiatry, IMS and SUM Hospital, Bhubaneswar. His therapy continued for 4 weeks with regular sessions. Following this he reported back for reevaluation. He was able to completely stop the SIB and there was a noticeable change in his gingival health with maintenance of fair oral hygiene. The residual gingival recession (Miller's Class II) was planned for correction with mucogingival surgery.

### 2.2. Case  2

**Figure 2:**
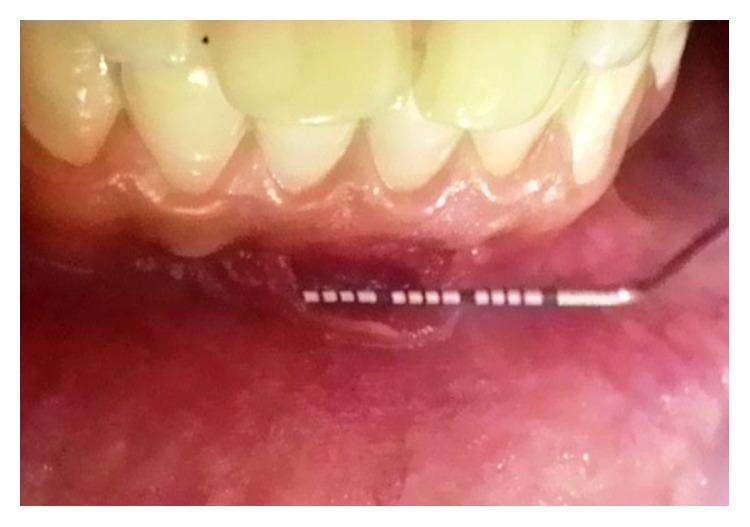
Case  2. Lacerated lesion due to matchstick picking involving the attached gingiva and alveolar mucosa in relation to teeth #32, #31, and #41.

A 28-year-old male reported to us with the chief complaint of sharp pain in mandibular anterior region 2 days back. He was apparently alright 2 months back when he noticed small lesion developing in the area of chief complaint. The lesion slowly increased in size and was associated with dull pain which aggravated 2 days back manifesting as sharp pain which led him to report to the department. Intraoral examination revealed a lacerated lesion involving the attached gingiva and alveolar mucosa in relation to mandibular incisors (Figure 2) measuring 9 mm × 4 mm × 2 mm. There was no exposure of bone in the affected area. He had a history of habit of picking the mandibular anterior area with a matchstick which led to this laceration. He had a history of suspension from his job a year back and admitted to being depressed for the past 6 months owing to his unemployment. On further probing, it was apparent that his picking habit seemed to have developed alongside his depression. He maintained fair oral hygiene with no evidence of any other dental discrepancy. He appeared to be well-built and healthy with no other relevant medical history. On referral to the Department of Psychiatry he was diagnosed as a case of depression with an adjustment disorder establishing that a diagnosis of gingivitis artefacta major was made based on his history and clinical findings.

#### 2.2.1. Management

Oral and behavioral management plan was explained to the patient and a written consent was obtained on his acceptance. The affected area was cleaned gently with cotton gauze soaked in normal saline to remove any accumulated food particles. He was prescribed with an antimicrobial mouthwash (HiOra mouthwash-regular, Himalaya Drug Company, Bangalore, India) and an anesthetic gel containing 20% Benzocaine (Mucopain, ICPA Health Products Limited, Ankleshwar, India) for symptomatic relief. Further, he was asked to refrain from picking in the concerned area.

Further, he was put on CBT for 6 weeks for behavioral management. Following CBT he was able to quit his SIB and reported to our department with remission of his oral lesion. No further periodontal correction was required and no recurrence was observed on follow-up of 3 months.

### 2.3. Case  3

**Figure 3:**
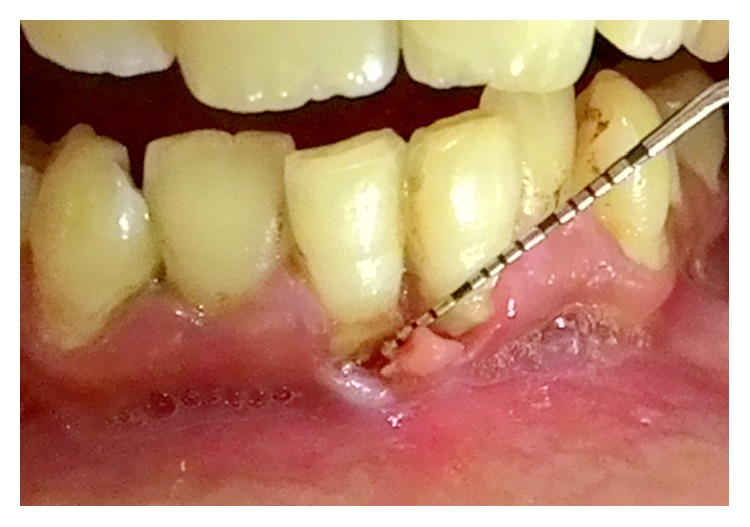
Case  3. Gingival recession due to scratching with fingernail and sharp objects in relation to teeth #31 and #41.

A 27-year-old male presented to us with the chief complaint of gnawing pain and sensitivity in mandibular anterior region 1 week back. On intraoral examination, gingiva appeared to be detached from the underlying tissues with evidence of gingival recession extending beyond the mucogingival junction in relation to mandibular central incisors (Figure 3). There was gingival erythema and profuse bleeding while probing with presence of local factors like plaque and calculus in the same area. Also, he had generalized plaque and calculus deposits suggesting inadequate oral hygiene maintenance. There was no evidence of any other dental discrepancy. He appeared anxious and on asking admitted to have been picking the affected area with his fingernail and other sharp objects like toothpick, fork, and so forth for the past one year. He also revealed to have been estranged from his spouse one year back for which he was depressed. His medical history was noncontributory and he was not currently under any medications. On psychiatric consultation, his depression and SIB were confirmed and a diagnosis of gingivitis artefacta major was made based on his history and clinical findings.

#### 2.3.1. Management

Periodontal and behavioral management plan was explained to the patient and a written consent was obtained on his acceptance. SRP was performed and oral hygiene instructions were given. He was prescribed desensitizing mouthwash (HiOra-K, Himalaya Drug Company, Bangalore, India) to be used twice daily for two weeks.

His behavioral therapy continued for two months where he underwent CBT in regular sessions. He, however, refused any surgical intervention and did not turn up for the subsequent appointment. In spite of several attempts, he could not be followed up.

## 3. Discussion

Oral lesions such as gingivitis artefacta are difficult to diagnose due to their similarity of clinical presentation with other oral lesions that may not have any underlying behavioral component. Differential diagnosis of gingivitis artefacta may include physical or chemical injury of gingiva, aphthous ulcer, and gingival recession. In addition, several syndromes and congenital conditions have been associated with SIB which includes Lesch-Nyhan syndrome, Cornelia de Lange syndrome, Tourette's syndrome, Rett syndrome, XXXXXY syndrome, XYY syndrome, and autism [9].

Traumatic injuries to the gingiva which are self-inflicted with an underlying emotional component pose diagnostic and management challenge: firstly, due to their similarity with those that are not self-inflicted and without any psychological basis and, secondly, owing to potential recurrence when they are unrecognized and undertreated.

Cases described here illustrate the essentiality of behavioral intervention in addition to periodontal procedures in the management of gingivitis artefacta major. These lesions are a manifestation of SIB. Self-injury can occur across the spectrum of age, gender, educational background, ethnicity, socioeconomic status, and religion. It is more common among adolescent females [6]. However, all the cases reported here were males in their twenties with emotional disturbances and developed self-inflicted injuries.

Oral self-injury may be functional or organic; while functional self-injuries are deliberate attempts aimed at attracting attention, organic self-injuries are inflicted unconsciously, in a compulsive manner and with no specific intent [11]. The diagnosis of emotional disturbances associated with SIB is generally ascertained by a psychiatric professional. This behavior can be a symptom of several psychiatric illnesses: personality disorders, bipolar disorder, major depression, and anxiety disorders (e.g., obsessive-compulsive disorder), as well as psychoses such as schizophrenia and also intellectual disability [1, 12].

Association of SIB with emotional component necessitates the need for behavioral intervention such as problem solving therapy, systemic behavioral family therapy, nondirective supportive therapy, and dialectic behavior therapy [10], as required in these cases. CBT is a type of psychotherapy that helps people to change unhelpful or unhealthy thinking habits, feelings, and behaviors in individuals experiencing a wide range of mental health difficulties [13]. It is an amalgamation of two therapies: cognitive therapy and behavior therapy. While the cognitive therapy focuses to change the way the person thinks about the issue that is causing concern, behavioral therapy is engaged to impart personal techniques to alter their behavior.

In the context of presented cases, it is noteworthy that basic periodontal therapy only cleared the local deposits resulting in an instantaneous improvement in gingival health. However, correction and long term stability of gingival recession which requires surgical procedure demands rendering the person free from SIB. Therefore, CBT may be a part of phase 1 periodontal therapy (if required) because it directly deals with patient motivation.

Management of patients with SIB injuries is usually difficult because of their lack of compliance and communication. In the cases presented here, psychiatric referrals were made after SRP. All the patients accepted the need for a behavioral intervention after presentation of facts and counseling. While the first two cases reported the benefits of CBT, in the third case, though he underwent the CBT and completed it, the patient did not report back to us for periodontal follow-up. His refusal to surgical therapy may have been due to either resolution of his chief complaint or fear of surgery.

Behavioral intervention in the form of CBT has been previously reported in periodontics with significant reduction in distress and anxiety and changed irrational beliefs and expectations [14]. Jönsson et al. [15] found that a CBT was an important predictor of oral hygiene behavioral change. Similar improvement was also observed with our patients after CBT.

## 4. Conclusions

Cases discussed in this report highlight the pivotal role of in-depth history-taking, which reveals underlying emotional involvement in a seemingly routine dental complaint and exposes self-inflicting behavior, necessitating behavioral intervention in addition to periodontal procedures in the management of lesions such as gingivitis artefacta major.
